# Pre-schoolers’ tooth brushing behaviour and association with their oral health: a cross sectional study

**DOI:** 10.1186/s12903-021-01643-8

**Published:** 2021-06-02

**Authors:** Iqra Muhammad Khan, Shani Ann Mani, Jennifer Geraldine Doss, Mahmoud Danaee, Lydia Yi Li Kong

**Affiliations:** 1 Department of Paediatric Dentistry and Orthodontics, Faculty of Dentistry, University of Malaya, 50603 Kuala Lumpur, Malaysia; 2 Department of Community Oral Health and Clinical Prevention, Faculty of Dentistry, University of Malaya, 50603 Kuala Lumpur, Malaysia; 3 Department of Social and Preventive Medicine, Faculty of Medicine, University of Malaya, 50603 Kuala Lumpur, Malaysia

**Keywords:** Dental hygiene, Pre-school children, Oral health status, Toothbrushing behaviour

## Abstract

**Background:**

Toothbrushing is an important yet neglected behaviour that affects the oral health of preschool children. Little is reported on parental supervision, an essential aspect of routine effective toothbrushing in this age group. The aim of this study was to evaluate pre-schoolers’ toothbrushing behaviour including parental involvement and its association with their oral health.

**Methods:**

This was a cross-sectional study. A total of 92 preschool children (4–6 years) were invited to participate with their parents/guardians. Nine parameters of toothbrushing behaviour were assessed from parental responses (questionnaire) and observation of child and parents/guardians (video recording). Oral examination included recording plaque, gingival and dental caries indices. BORIS software was used to assess toothbrushing parameters and Smart PLS was used to perform association with a second-generation multivariate analysis to create models with and without confounding factors.

**Results:**

Girls were slightly more (53%) than boys (47%). Children aged 4 years were slightly more in number (38%), followed by 6-year-olds and 5-year-olds. Nearly, 90% parents had tertiary education and 46% had more than 2 children. Differences were recorded in the reported and observed behaviour. Thirty-five percent parents/guardians reported using pea-size toothpaste amount but only 28% were observed. Forty percent reported to brush for 30 s–1 min, however 51% were observed to brush for 1–2 min. Half the children were observed to use fluoridated toothpaste (F < 1000 ppm) under parental supervision (11%). The mean (SD) plaque score reduction after toothbrushing was 10.80 (2.46), mean pre-brushing plaque score was 90.3 (10.2), mean gingival index was 0.89 (0.65) and mean dental caries status (ICDAS_(1–6)_) was 18.87 (12.39). Toothbrushing behaviour in terms of toothbrushing technique, duration, pattern and frequency, toothbrush type and grip type, toothpaste type and amount, post-brushing mouth rinsing and parental involvement contributed significantly to plaque score change (86%), dental caries status (73%), gingival index (66%) and pre-brushing plaque score (31%). The significant confounding variables had a small influence on oral health of preschool children.

**Conclusions:**

Preschool children’s toothbrushing behaviour was inadequate while their oral health was poor, with a significant association between the two parameters.

## Background

Oral health is an important aspect of general health [1], with preschool children at high-risk for developing oral diseases [2]. Factors associated with oral disease include poor toothbrushing routine, poor dietary habits, low socioeconomic status and concurrent oral conditions [3]. Improper oral health care and altered dietary patterns interrupt the microbial homeostasis within the oral cavity, promoting biofilm formation responsible for oral diseases such as dental caries and gingivitis. Effective toothbrushing disturbs this biofilm formation and prevents oral disease [4].

According to recommendations, toothbrushing should start with the eruption of first primary tooth [5]. A supervised toothbrushing of preschool children should be done twice a day, for two minutes with appropriate size soft bristled toothbrush (15–19 mm head size) and pea-sized fluoridated toothpaste [6]. Post brushing mouth rinsing should be kept to a minimum to retain the fluoride effect in the oral cavity [7].

Pre-schoolers’ have inadequate toothbrushing behaviour because they did not follow toothbrushing recommendations [8, 9] and were not supervised by their parents during toothbrushing [10]. Moreover, a recent study on Malaysian school children revealed poor oral health knowledge, attitudes and practices [11].

Despite multiple interventions, the oral health status among preschool children remains poor [2, 12]. Consequently, it is imperative to investigate their toothbrushing behaviour and routine oral hygiene practices. Therefore, the aim of this study was to evaluate the toothbrushing behaviour of a group of Malaysian preschool children and determine the association with their oral health status. The study hypothesized that toothbrushing parameters were associated with the oral health status of preschool children.

## Methods

### Study design and location

This cross-sectional study is reported according to the “Strengthening the Reporting of the Observational Studies in Epidemiology (STROBE)” statement. This single visit study consisted of 92 participants that were drawn from the outpatient paediatric dental clinic, Faculty of Dentistry, University of Malaya using convenience sampling technique until the required sample size was achieved. A latest version of G-Power sample size calculator [13] estimated the sample size maintaining the power at 0.90 (90%) and significance level of 0.05, using a correlation value of 0.3 between toothbrushing duration and plaque score from a previous study [9]. The study participants were healthy (free from oral and systemic diseases), Malaysian preschool children (4–6 years) accompanied by their parents/guardians who were familiar with English and/or local language. The data collection was completed in one year (March 2018–19). Ethical approval for this study was obtained from the University Medical Ethics Committee [DF CD1707/0039(L)]. All procedures performed were in accordance with the ethical standards of the institutional committee and with the 1964 Helsinki declaration and its later amendments or comparable ethical standards.

### Conduct of the study

Written informed consent was obtained from parents of eligible children and parents/guardian interested to participate in the study. Parents/guardians completed a questionnaire (described below). Subsequently, the children were examined for their gingival status and pre-brushing plaque score. Children were then invited to brush their teeth accompanied by their parents/guardians at a setup that consisted of a sink, a mirror, disposable cup, a stool for participant's convenience and a range of commonly available toothpastes.

At the sink, each child was provided with age-related child toothbrush. The children selected a toothpaste of their choice, based on the one used at home, from a variety of four fluoridated (one F > 1000 ppm and three F < 1000 ppm) and one non-fluoridated brands. A video recorder mounted onto a tripod stand was placed behind the mirror to discreetly record the toothbrushing. Parents/guardians were encouraged to participate in the toothbrushing session as they did at home. In order to prevent bias, only parents/guardians who gave consent were informed about the video recording and the researcher left the room during the toothbrushing session. Following the toothbrushing session, participants were re-examined for post-brushing plaque score. A complete oral prophylaxis was done prior to assessing the dental caries status. Finally, the children and parents were taught a standardized toothbrushing technique.

### Study tools and scoring criteria

#### Questionnaire (Parental response)

A self-administered questionnaire by parents/guardian was used to assess the oral health behaviour of their child. It comprised four sections: Section I (four items) and II (five items) were demographic/socioeconomic status of parents and child respectively; section III and IV assessed the child’s dietary habits and oral hygiene practices (toothbrushing behaviour) (10 items) respectively. First three sections were considered confounding factors affecting oral health. The questionnaire was adapted from a previous study [14] and then translated to Bahasa Melayu (local language) by a local translator. Parents/guardians responses in the Bahasa Melayu were translated back to English.

#### Gingival status

The primary index teeth (55, 52, 64, 75, 72, 84) were evaluated with Loe and Silness gingival index (1963) [15].

#### Plaque score

The pre and post brushing plaque scores were charted according to the 'The Plaque Control Record’ [16] criteria following the application of a plaque disclosing dye (Mira-2-tone).

#### Dental caries

Full mouth charting was done after oral prophylaxis using the International Caries Detection and Assessment System (ICDAS) [17]. The score was later converted to decayed filled surfaces (dfs); dfs_(1–6)_ (total caries), dfs_(1–3)_ (enamel caries) and dfs_(4–6)_ (dentine caries) for analysis.

#### Parental guidance

Parental guidance scoring criterion was developed after the observation and assessment of the parents/guardian's involvement in their children toothbrushing session during the pilot study and enlisted as follows:

**Table:** 

Score 0	No involvement by the parents/guardians while the child was brushing his/her teeth
Score 1	Parents/ guardians observed while the child was brushing his/her teeth independently, but gave no further input
Score 2	Parents/guardians provided verbal advice while the child was brushing his/her teeth independently
Score 3	Parents/guardians brushed their child’s teeth, not permitting them to brush independently
Score 4	Parent/guardians brushed their child’s teeth, after the child attempted to brush independently
Score 5	Parent/guardians observed, used a verbal and hands-on approach to assist their child during toothbrushing

#### Video recording (Observed)

Children’s toothbrushing behaviour was analysed using a software, BORIS (Behavioural Observation Research Interactive Software) [18] to extract the following toothbrushing parameters:

- Toothpaste type: The toothpaste selected by the child/parent based on the fluoride content.
- Toothpaste amount: The toothpaste applied on the toothbrush by the child/ parent was categorized according to toothpaste length on brush (full, half, pea-size, smear).
- Toothbrush grip: The type of grip on the handle of the toothbrush by the child.
- Toothbrushing duration: Time frame between first placement of toothbrush on the teeth till it was removed from the mouth.
- Toothbrushing technique: Based on the observed directions of the toothbrush strokes.
- Toothbrushing pattern: Based on systematic vs non-systematic approach to toothbrushing.
- Post-brushing mouth rinsing: The number of times a child rinsed their mouth after completion of toothbrushing.
- Parental guidance: The parents/guardians were scored for their involvement in the tooth brushing session according to the criteria.

#### Partial least square structural equation modelling (PLS-SEM)

An advanced statistical tool, Smart PLS version 3.2.9 was used to determine the association between toothbrushing behaviour and oral health status of preschool children [19]. A second-generation multivariate analysis was performed to create two models, first without confounding variables and second with confounding variables. Each model had an outer/ measurement and inner/structural model. The outer/measurement model explained the influence of each factor (e.g. electric toothbrush) on its respective latent variable (e.g. toothbrush type). The number denoted the strength of the contribution, with a higher number indicating a stronger contribution. The multicollinearity determined by Variance Inflation Factor (VIF) for all the variables contributing to the toothbrushing behaviour was calculated to check for highly correlated variables. The variables of outer model were divided into formative constructs; group of factors contributing to form the respective latent variable and reflective constructs; factors that are formed by their respective latent variable. The inner/structural model represented the relationship between toothbrushing behaviour (IV) and oral health status (DV) and was explained in terms of coefficient of determination, path analysis and bootstrapping. The coefficient of determination (R^2^) was interpreted as the proportion of the variance in the dependent variable that is predicted by the independent variable. The path analysis (β) determined the causal linkage between toothbrushing behaviour and oral health status and bootstrapping; which is a test for estimation of sampling distribution using random sampling method (*p*-values).

### Calibration of study tools and statistical analysis

Content validation of questionnaire was performed by a panel of four experts from the department of paediatric dentistry and community dentistry. The questionnaire was pretested on 10 parents/guardians (not involved in main or pilot study), resulting in minor changes in two sections of questionnaire (5 questions). Questionnaire reliability (test–retest coefficient) was 0.7–0.8 [20].

A pilot study was conducted on 15 preschool children and their parents, during which the inter and intra-rater reliability testing of all the tools and indices were done. The inter-rater reliability of indices and observed parental behaviour in the videos were tested by two raters; a trained postgraduate dental student and paediatric dental specialist, and intra-rater reliability was conducted by repeated readings by a trained postgraduate dental student. The toothbrushing videos recorded during the pilot study were calibrated in a similar way between two raters; a trained post-graduate student and software expert, followed by intra-rater calibration by the post-graduate student. The two-way intraclass correlation coefficient values (ICC) calculated for inter and intra-rater calibration for the various tools and indices are as follows; gingival index was 0.87 and 0.97, pre-brushing plaque score was 0.85 and 0.97, post-brushing plaque score was 0.78 and 0.99, dental caries charting was 0.88 and 0.9 and video recording was 0.7 and 0.8 respectively. The kappa score for parental guidance was 0.8 [21]. All data was analysed using the latest version of SPSS version 26 [22].

Descriptive statistics are presented as frequency and percentage for toothbrushing behaviour and mean (SD) for oral health status. The association between the toothbrushing behaviour (independent variable; IV) and oral health status (dependent variable; DV) was determined by Smart PLS version 3.2.9 [19]. The confounding variables showing a significant correlation with oral health status (DV) based on the SPSS data analysis were included. A confidence interval of 95% and a *p*-value of 0.05 were set.

### Confounding factors

Dietary habits and socioeconomic status of the child were added to the model as confounding factors and their results (with and without confounding factors) were compared.

## Results

### Demographic characteristics of preschool children

Of 92 participants, 49 were girls. Two-thirds of participants were Malay (66%), while all other ethnicities (Chinese, Indian and other) accounted for 34% of the sample. The age distribution of the sample was 4 year olds (38%), 5 year olds (29%) and 6-year-olds (33%). Majority (90%) parents/guardians had tertiary level education. The response rate was 79% with a dropout of 25 participants. The flow chart of the data collection procedure with the details of the participants at every stage of the study is given below.



There were differences between the reported and observed results of toothbrushing behaviour. The amount of toothpaste reportedly used by slightly more than one-third of parents was pea-size (35%) whereas only 28% were observed to do so. Many preschool children reported to brush for 30s–1 min (40%) however only 14% were observed. Mostly, their toothbrushing duration was 1–2 min (51%) on observation. About 74% reported to use fluoridated, toothpaste (F < 1000 ppm) however, only 50% were observed to do so. About 52% of parents/guardians reported guiding their children toothbrushing occasionally but on observation only 11% were verbally and practically involved. Slightly less than half (46%) of parents were observed to be totally uninvolved during the toothbrushing session. Details of reported and observed toothbrushing behaviour are shown in Table 1.

**Table 1:** Reported and observed toothbrushing behaviour (IV) of preschool children

Variables (n = 92)		Parental/guardian response n (%)	Observation n (%)
Age at which parent–child initiated toothbrushing	< 6 months	9 (9.8)	–
	6 month–1 year	16 (17.4)	
	1–2 years	37 (40.2)	
	2–3 years	7 (7.6)	
	3–4 years	23 (25)	
Toothbrushing frequency	Once a day	8 (8.7)	–
	Twice a day	46 (50)	
	> 2 times a day	11 (12)	
	Once in 2–3 days	10 (10.9)	
	Rarely	17 (18.5)	
Toothbrush type use	Electric toothbrush	21 (22.8)	–
	Manual toothbrush	62 (67.3)	
	Finger or other object	9 (9.8)	
Frequency of toothbrush change	Once in 15 days	15 (16.3)	–
	Once in month	4 (4.3)	
	Every 2–3 months	24 (26.1)	
	Once bristles frayed out	47 (51.1)	
	Has not changed yet	2 (2.2)	
Toothpaste amount	Smear	28 (30.4)	43 (46.7)
	Pea	32 (34.8)	26 (28.3)
	Half length	9 (9.8)	14 (5.2)
	Full length	23 (25)	9 (9.8)
Toothbrushing duration	< than 30 s	13 (14.1)	18 (19.6)
	30 s–1 min	37 (40.2)	13 (14.1)
	1–2 min	35 (38.0)	47 (51.1)
	> 2 min	7 (4)	14 (15.2)
Toothpaste type	Fluoridated (F > 1000)	11 (12)	28 (30.4)
	Fluoridated (F < 1000)	74 (80.4)	46 (50)
	Non-fluoridated	7 (7.6)	18 (19.6)
Parental guidance (questionnaire)	Yes, everyday	40 (43.5)	–
	Yes occasionally	48 (52.2)	
	No	4 (4.3)	
Toothbrush sharing with siblings	No	77 (83.7)	–
	No, because only child	10 (10.9)	
	Yes	4 (4.3)	
Visit to dental clinic	Yes, for dental check-up	32 (34.8)	–
	Yes, for tooth ache	28 (30.4)	
	No	32 (34.8)	
Toothbrushing technique	Horizontal	–	38 (41.3)
	Vertical		21 (22.3)
	Rotatory motion		15 (16.3)
	Other method		18 (19.6)
Toothbrushing pattern	Systematic	–	45 (48.9)
	Non-systematic		47 (51.1)
Toothbrush grip (Child grip) (n = 78)	Oblique	–	21 (26.9)
	Distal oblique		27 (34)
	Precision		11 (14.10)
	Power		14 (15.2)
	Spoon		5 (6.4)
Post-brushing mouth rinsing	Do not rinse	–	5 (5.4)
	Once		67 (72.8)
	Multiple times		20 (21.7)
Parental guidance during tooth brushing (observation)	0	–	42 (45.7)
	1		9 (9.8)
	2		8 (8.7)
	3		17 (18.5)
	4		6 (6.5)
	5		10 (10.9)

Table 2 shows the details of oral health status of participants. Although a mean plaque score reduction of 10.9 (2.46) was noted after toothbrushing, the overall score (pre and post brushing plaque scores) remained poor (> 35%). Overall, 30.4% had healthy gums, 50% had mild gingivitis and 19.6% had moderate gingivitis. Only 4.4% tooth surfaces had no caries and mean dental caries (dfs_(1–6)_) was 18.8 (12.3).

**Table 2:** Oral health status of preschool children

Variable	Grading n(%)		Mean	Standard deviation
	Severity/category	n(%)		
Pre-brushing plaque score^a^	Poor (> 35%)	92 (100)	90.3	10.2
Post-brushing plaque score^a^	Poor (> 35%)	92 (100)	79.5	9.7
Plaque score change	–		10.8	2.4
Gingival Index^b^	No gingivitis	28 (30)	0.8	0.6
	Mild gingivitis	46 (50)		
	Moderate gingivitis	18 (19.6)		
Dental caries status^c^	dfs_(1–3)_	54 (58.7)	8.1	9.3
	dfs_(4–6)_	34 (37)	10.5	15.5
	dfs_(1–6)_	88 (95.6)	18.8	12.3

^a^O’Leary’s and drake method (88 tooth surfaces)

^b^Loe and Silness gingival index (28 tooth surfaces)

^c^ICDAS (88 teeth)

### Partial least square structural equation modelling (PLS-SEM)

**Fig. 1:**
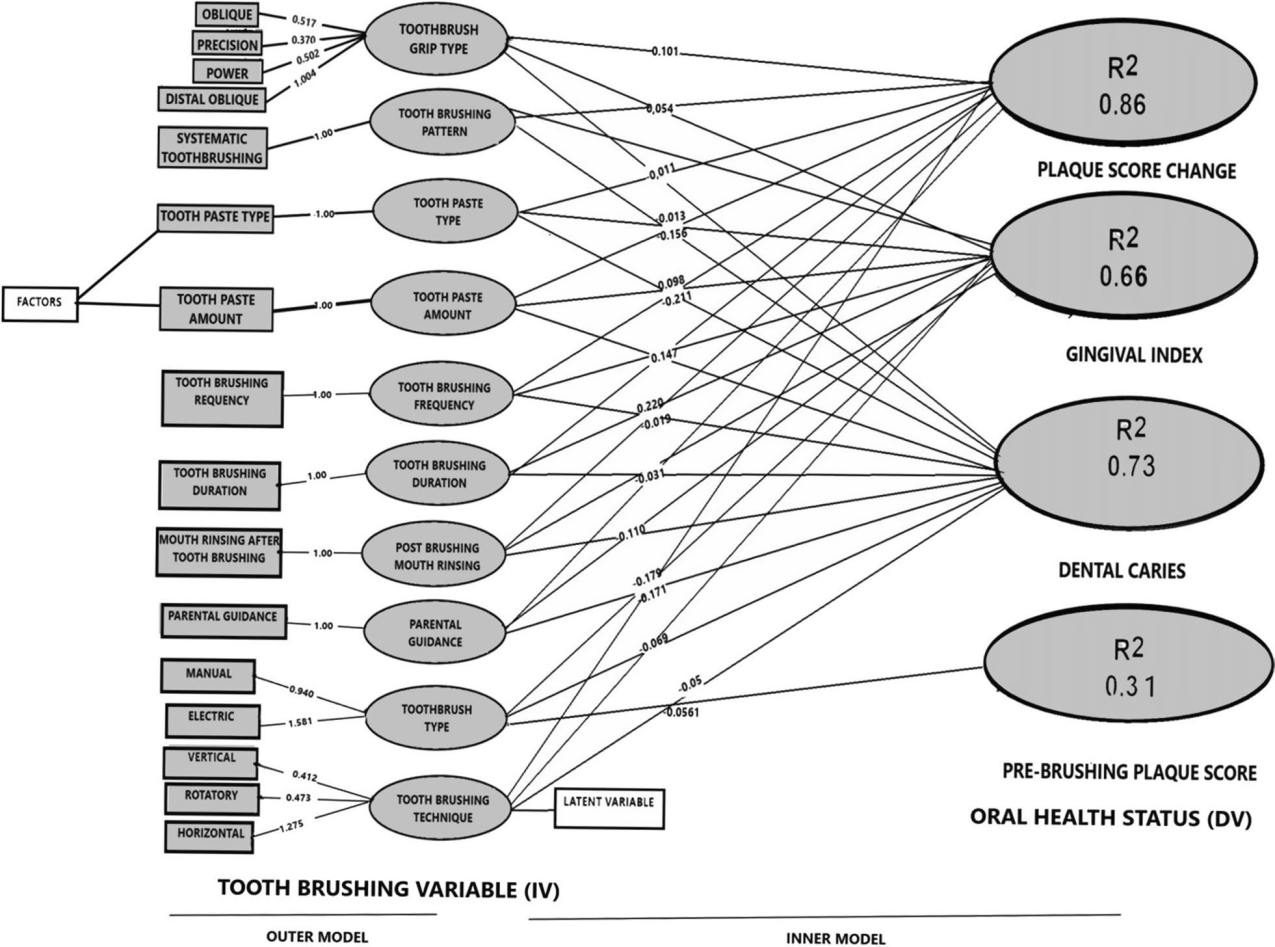
A path model showing association between IV and DV

Figure 1 shows the multivariate analysis depicting the association between toothbrushing behaviour and oral health status of preschool children using Smart-PLS software.

Outer model: This model explains the contribution of each factor on its respective latent variable (IV) using outer weight. All IVs (refer to Table 3) had low VIF value (< 3) and thus individually were able to explain the preschool children’s toothbrushing behaviour independently. When comparing different indicators of individual IV, electric toothbrush (1.581) contributed more to the toothbrush type than a manual toothbrush (0.94); distal grip oblique (1.004) contributed more to the toothbrush grip type than others; horizontal technique contributed the most (1.275) to toothbrushing techniques; systematic toothbrushing sequence contributed more than non-systematic toothbrushing sequence. In terms of toothpaste, fluoridated toothpaste (F > 1000 ppm) and smear sized amount contributed more than other types. Brushing more than twice a day for more than 2 min was better than other options, while minimum post-brushing mouth rinsing contributed more than multiple mouth rinsing. Parental guidance in terms of verbal and hands-on participation was much better than other options.

**Table 3:** Association between toothbrushing behaviour and oral health status depicted by partial least square model^a^

No	Toothbrushing variables (IV)	Oral health status(DV)	β	SE	t value	P Values
1	Toothbrush type	Plaque score	− 0.56	0.06	8.94	**<0.001**
		Gingival index	− 0.24	0.07	3.42	**<0.001**
		Dental caries	− 0.07	0.07	0.94	0.35
2	Toothbrush grip type	Plaque score	0.10	0.06	1.67	0.10
		Gingival index	− 0.06	− 0.18	2.64	**0.01**
		Dental caries	− 0.06	0.07	0.96	0.34
3	Toothpaste type	Plaque score	0.15	0.07	2.34	**0.02**
		Gingival index	− 0.06	0.11	0.50	0.62
		Dental caries	− 0.21	0.10	2.10	**0.05**
4	Toothpaste amount	Plaque score	− 0.01	0.06	0.23	0.82
		Gingival index	− 0.03	0.07	0.46	0.65
		Dental caries	− 0.18	0.06	3.32	**<0.001**
5	Toothbrushing frequency	Plaque score	0.10	0.06	1.69	0.10
		Gingival index	− 0.25	0.09	2.81	**0.01**
		Dental caries	− 0.20	0.07	2.81	**0.01**
6	Toothbrushing duration	Plaque score	0.15	0.05	2.86	**0.01**
		Gingival index	− 0.02	0.09	0.23	0.83
		Dental caries	− 0.24	0.09	2.77	**0.01**
7	Toothbrushing technique	Plaque score	0.18	0.05	3.85	**<0.001**
		Gingival index	− 0.25	0.12	2.04	**0.05**
		Dental caries	− 0.02	0.06	0.25	0.80
8	Toothbrushing sequence	Plaque score	0.05	0.07	0.79	0.43
		Gingival index	− 0.01	0.11	0.10	0.92
		Dental caries	− 0.16	0.08	2.03	**0.05**
9	Mouth rinsing	Plaque score	0.22	0.06	3.92	**<0.001**
		Gingival index	− 0.03	0.09	0.36	0.72
		Dental caries	− 0.11	0.06	1.73	0.09
10	Parent involvement	Plaque score	0.34	0.05	6.63	**<0.001**
		Gingival index	− 0.20	0.09	2.11	**0.04**
		Dental caries	− 0.17	0.07	2.28	**0.03**

Bold is statistically significant where p < 0.05

^a^Without confounding factors

Inner model: This model explains the association between toothbrushing behaviour (IV) and oral health status (DV) of preschool children. The relationship is presented in terms of coefficient of determination R^2^ (variance), path coefficient (causal linkage between toothbrushing behaviour and oral health status) and bootstrapping (*p*-values) as shown in Table 3. The R^2^ value for plaque score change was 0. 860. This indicated that toothbrushing technique and duration, post-brushing mouth rinsing and parental guidance significantly explained 86% variance in children’s plaque score change. A variation of 31% in pre-brushing plaque score was highly explained by toothbrush type (R^2^ 0.315). Similarly, toothbrushing frequency, toothbrush type, toothbrush grip type, toothbrushing technique and parental guidance significantly explained 66% variance in the children's gingival index (R^2^ 0.66). Toothbrushing frequency, toothbrushing pattern, toothbrush type and toothpaste amount and parental guidance explained 73% variance in their dental caries status (R^2^ 0.73).

Confounding factors: Upon inclusion of confounding factors (dietary factors and socioeconomic status) into the model, a small effect was noted on the variance (0.02–0.03) of children’s gingival index (0.69), pre-brush plaque score (0.33) and dental caries (0.76) however no change was observed in the variance of plaque score change.

## Discussion

Early Childhood Caries is a global health problem affecting almost 50% of the population and varies widely between continents [23]. Moreover, high traces of visible plaque were found on tooth surfaces of preschool children suffering from oral diseases, signifying the importance of oral hygiene maintenance for good oral health [24, 25]. In this study, the reported toothbrushing behaviour of preschool children was different from the observed toothbrushing behaviour in terms of toothpaste type and amount, toothbrushing frequency, duration, technique and pattern, mouth rinsing and parental guidance. However, on comparison both (reported and observed) tooth brushing behaviours were unsatisfactory. The reported behaviour was similar to the findings of another Malaysian study on 4 to 6-year-old’s in terms of toothbrushing frequency (> 50%), use of fluoridated toothpaste (92%), children toothbrush (90%) and pea-sized toothpaste amount (> 30%) [26]. Factors including dietary habits and socioeconomic status also affected the oral health status of preschool children [2]. Malaysian parents/guardians had adequate oral heath knowledge, but poor practice which led to compromised oral health of their children [27, 28]. Hence, knowledge is not necessarily translated into good practice, perhaps due to lack of in-depth knowledge of toothbrushing aspects. In our study, oral health status of preschool children was poor, similar to other Asian populations [29]. The overall prevalence of dental caries reported in this study was higher than in the national survey (71.3%) [2].

An advanced statistical analysis (second generation multivariate analysis) to test the association between IV and DV was performed (Fig. 1). The model depicted a better oral health status (plaque score, gingival index and dental caries status) in children using the electric toothbrush compared to others. Similar findings of higher plaque score change was noted for electric toothbrush, when compared to manual toothbrush possibly because electric brushes are less technique sensitive requiring less dexterity [30]. The preference of toothbrush grip type among Malaysian preschool children was similar to that of Indian children, which was distal oblique grip type followed by oblique, power, precision and spoon type [31]. Distal oblique grip type resulted in better plaque removal than other grip types [32]. However, in our study, toothbrush grip type was not significantly associated with plaque score but instead only significantly associated with gingival status. Majority of children were observed to use fluoridated toothpaste similar to that reported in another Malaysian study [33]. However, the trend of using non-fluoridated toothpaste has remained at 8–10% in both these studies [33]. When compared to our study, the use of fluoridated toothpaste among preschoolers, was reportedly more in Trinidad (80%) [34], and less in Hispanics (71%) [35]. Higher fluoride containing toothpaste (F > 1000 ppm) had a significant effect on dental caries status and plaque score change in the present study [36]. This may also explain why children in our study using full-length toothpaste had lesser dental caries. However, pea-sized toothpaste amount is routinely recommended to avoid side effects such as fluorosis in children who inadvertently swallow the toothpaste [5]. The percentage of preschool children in the present study reportedly using  pea-sized toothpaste (19%) was more than that reported previously [33]. However, those observed to use pea-size was less than the reported number.

Toothbrushing twice a day or more was associated with better oral health status (dental caries and gingival health) of preschool children [37]. In our study, sixty two percent of preschool children brushed twice a day or more, which was slightly more than the previous study on Malaysian pre-schoolers (59.4%) [14], but less than that reported by Swedish investigators [37]. In our study better gingival and dental caries status were recorded with more frequent toothbrushing, understandably due to the routine biofilm disturbance. Compared to recommendations, shorter toothbrushing duration was observed in other studies among pre-schoolers [35, 38]. In this study increased toothbrushing duration was observed (51%), speculating that children attempted to remove the plaque disclosing dye which they could visualise on their teeth. The horizontal technique of toothbrushing was preferred in this study, similar to another study [8], explicably due to the lack of manual dexterity in this age group. Toothbrushing in a systematic way ensures increased plaque removal [39]. The children observed to brush in a non-systematic manner in our study had poorer oral health, although the association was only significant with gingival status, not plaque score. Routine non-systematic toothbrushing will more likely manifest as localised gingivitis. Minimal mouth rinsing (with water) after toothbrushing is currently recommended to retain the antiplaque effect of fluoride in oral cavity for a longer period [7]. We observed that majority rinsed once after toothbrushing, although they rinsed multiple times during the course of toothbrushing. The effect of multiple mouth rinsing midway during toothbrushing will most likely reduce the fluoride effect of toothpaste, however evidence on this is lacking.

The necessity for parental supervision among pre-schoolers is underpinned in the literature [38, 40], yet parental supervision during toothbrushing was not evaluated in previous studies. We observed parents’ supervision of their children’s toothbrushing ranged across five categories, of which 46% were not involved at all. Better oral health status was observed in preschool children who brushed their teeth under increasing parental supervision. Parents/guardians adaptive behaviour management strategies during toothbrushing was associated with better children oral health status [38]. A study of mother’s oral health behaviours in Malaysia also reported that a greater percentage (46%) of parents/guardians were uninvolved during their children toothbrushing session [26]. Plausible reasons for inadequate toothbrushing supervision include parental inability to provide individual attention to their children especially in large families, working mothers or inadequate awareness among parents on the need for supervision.

This study used innovative tools and software that were not used in the field of dentistry previously (e.g. PLS and BORIS). Moreover, to the best of our knowledge, this was the first study conducted on the toothbrushing behaviour on Malaysian pre-schoolers. Several studies have been conducted on parental guidance during pre-schoolers toothbrushing. However, the present study has added value by being one of the first to grade parental guidance according to their extent of involvement during their children’s toothbrushing [38, 40]. Additionally, recording pre and post brushing plaque scores provided a better understanding for parents/guardian about the role of toothbrushing in effective plaque removal. This study can be used as baseline for studying toothbrushing behaviour of preschool children of other populations.

This study is not without limitations. Firstly, the relatively small sample size in our study could have affected associations between plaque score and some of the toothbrushing parameters, for example, toothbrush grip type, toothbrushing pattern and post brushing mouth rinse. Secondly, children’s attempt at removing the plaque disclosing stain on their teeth may have prolonged toothbrushing duration. Thirdly, daily time of oral examination differed among children and may have affected their pre-brushing plaque scores. Fourthly, the toothbrushing environment provided in the clinic differed from that in their homes and may have influenced their toothbrushing parameters. Lastly, children who used electric toothbrushes at home may have not been familiar with brushing using the manual toothbrushes given to them, thus affecting their post-brushing plaque scores. Future studies on a larger sample size and different populations are recommended for in-depth knowledge about oral health behaviour of pre-school children.

The present study emphasised the role of toothbrushing behaviour in the maintenance of preschool children’s oral health status. Adopting correct toothbrushing habits at an early age will become a lifelong habit that can reduce the chances of oral diseases in both dentitions. It is hoped that these findings will help raise awareness among clinicians, parents and policy makers in prioritising correct toothbrushing behaviour as part of the primary prevention program for this target group.

## Conclusion

Thus, with the outcomes of present study, the null hypothesis was rejected drawing the following conclusions:

1. The toothbrushing behaviour of preschool children was inadequate and their oral health was poor.
2. Toothbrushing behaviour is associated with oral health status of preschool children. Regardless of dietary habits and socioeconomic status, parental guidance during pre-school children’s toothbrushing is associated with better oral health status in their children.

## Data Availability

The datasets used and/or analysed during the current study are available from the corresponding author on reasonable request.

## Abbreviations

BORIS: Behavioural Observation Research Interactive Software
ICDAS: International Caries Detection and Assessment System
PLS: Partial least square
IV: Independent variable
DV: Dependant variable
